# Work-family conflict and the professional quality of life and their sociodemographic characteristics among nurses: a cross-sectional study in Tehran, Iran

**DOI:** 10.1186/s12912-022-01069-9

**Published:** 2022-11-01

**Authors:** Reza Biabani Dilmaghani, Baharam Armoon, Ladan Fattah Moghaddam

**Affiliations:** 1 Department of Nursing, Faculty of Nursing and Midwifery, Tehran Medical Sciences, Islamic Azad University, Tehran, Iran; 2 Social Determinants of Health Research Center, Saveh University of Medical Sciences, Saveh, Iran; 3 Department of Psychiatric Nursing, Faculty of Nursing and Midwifery, Tehran Medical Sciences, Islamic Azad University, Tehran, Iran

**Keywords:** Work-family conflict, Professional quality of life, Nurses

## Abstract

**Background:**

Nurses are exposed to work-family conflict (WFC) due to specific occupational conditions, such as exposure to patients and shift work, which can affect the professional quality of life (ProQoL). The aim of the present study was to determine the relationship between different levels of work-family conflicts and professional quality of life and their sociodemographic characteristics among the nurses in two hospitals in Tehran, Iran.

**Methods:**

This cross-sectional study included 234 nurses from two hospitals. Data were collected using a three-part questionnaire, including items related to nurses’ demographic characteristics, the WFC questionnaire, and the ProQoL questionnaire.

**Results:**

There was a significant positive correlation between conflict in terms of time and behavior with compassion satisfaction and between the three types of conflict with job burnout (p < 0.05). The results indicate that the WFC and ProQoL scores and the mean WFC were higher among women, people who had a disabled family member, and nurses who worked equal to or more than 175 hours a month. Regarding the dimensions of ProQoL, the mean compassion satisfaction was lower among people who lived with their parents, people who had a second job had a child younger than one-year-old, and a disabled family member had higher means of job burnout. Also, those who had younger children experienced less PTSD. Furthermore, the mean of WFC and burnout among single nurses was significantly lower than among married and divorced/widowed nurses. Additionally, the mean of WFC and burnout among nurses living in rental houses and nurses reporting the level of satisfying relationships with co-workers as ‘bad’ were significantly more than for other nurses.

**Conclusion:**

Stable mental and emotional conditions are of great importance for nurses to provide safe and quality services to their patients. The results also revealed that nurses’ compassion satisfaction and job burnout were associated with different types of conflict. The WFC of nurses is related to their ProQoL and affects their job satisfaction and burnout. Reducing conflict may improve the nurses’ satisfaction and thus improve patient care and healthcare services.

## Background

About one-third of an individual’s life is spent in the work environment [1], which can be a source of mental stress [2]. Work-family conflict (WFC) is defined by the existence of disparity between the work and family roles of individuals [3, 4], which consists of time-based WFC, strain-based, and behavior-based WFC [5]. WFC occurs because of the incompatibility between roles and conditions in the family with those in the workplace [6]; when professional duties reduce an individual’s time, commitment, and energy for their family, the result is a decrease in the individual’s ability to fulfill their family roles [7, 8].

Medical environments are among the most stressful working environments, and since nurses have the most direct contact with patients, they experience many different stressful conditions [9]. Undoubtedly, balancing professional lives and personal lives can enhance nurses’ mental power and influence their provisioned services, which in turn can result in improvements in the safety and satisfaction of patients. Moreover, that balance can increase family cohesion because of the nurses’ appropriate behavior and active presence in their families [10, 11].

The WFC among nurses may be due to the lack of work support, the work pressure of caring for severe state patients [12], the conflict between work and family roles of nurses and perceived conflicts with patients or other staff, and high workload, which may cause to job burnout [12]. In addition, dissatisfaction with wages, lack of opportunity for promotion, educational leave and insufficient staffing as well as resources, and absence of nurses’ participation in hospital management may cause high burnout levels [13–15]. In Iran, the health system was facing a shortage of nurses of about 1.3 nurses per 1000 in 2020, and the shortage is still increasing [16]. In other countries, such as the United States, the shortage of nurses was about 3 nurses per 1000 [17]. The concept of nursing shortage is associated with the scope of practice for nurses, the geographic characteristics of the activity, and the population and their requirements in that community [16]. In Iran, nursing shortage is one of the significant challenges and requires suitable strategies [18] since the shortage of nurses may increase the probability of physical and psychological stress, which may lead to burnout.

Professional quality of life (ProQoL) is another variable which can be influenced by the level of compatibility between work and life. ProQoL is related to people’s satisfaction with meeting their different needs in terms of resources, activities, and results and is achieved by playing the role of people in the workplace, which has three dimensions of satisfaction from empathy, job burnout, and post-traumatic stress caused by accident. It includes both positive and negative aspects simultaneously. The positive aspect is the satisfaction of a person in feeling empathy (compassion satisfaction), which is based on having effective interactions with patients based on understanding. However, the negative aspect is compassion fatigue, which consists of two dimensions: burnout and PTSD [19, 20].

Studies have shown that the level of WFC among nursing staff in Iran is not at the desired level [21, 22]. Alhani et al. revealed that about 60% of the nursing participants in their study experienced a high level of WFC [21]. Also, an investigation of married female nurses at 13 hospitals in Shiraz, Iran, showed that most of them experienced average to high levels of WFC [22]. Furthermore, studies demonstrated that WFC had adverse effects on nurses’ job satisfaction [7], burnout [23], and mental health [24].

Other studies have observed a direct relationship between ProQoL and nurses’ physical and mental health [4, 25, 26] and reported higher levels of dissatisfaction and burnout among nurses. A study revealed that ProQoL is one of the factors influencing nurses’ job stress and tolerance and has affected their performance [27]. A study of the quality of life among the staff of a trauma center showed that in terms of burnout and PTSD the quality of life was poor, while a moderate level of compassion satisfaction was also observed [28]. The findings of an investigation by Ariapooran [29] showed that 45.3% and 15.03% of the studied nurses experienced an unfortunate situation regarding empathic distress and burnout, respectively. To our knowledge, few studies in Iran have been carried out on WFC among nurses; no previous study has considered ProQoL among nurses that it might identify determinants of WFC and reduce WFC among nurses. Therefore, ProQoL and WFC have significant effects on nursing personnel management that may affect their performance in relation to healthcare responsibilities. The aim of the present study was to determine the relationship between different levels of work-family conflicts and professional quality of life and their sociodemographic characteristics among the nurses in two hospitals in Tehran, Iran.

## Methods

### Design and setting

This cross-sectional study was conducted in 2019 in two hospitals. Work-related conditions and workplace tensions are more prevalent in hospitals located in more populated cities [24]. These hospitals are also forced to provide support and medical services in critical situations, which intensifies the work pressure on nurses. Therefore, we randomly selected two hospitals from the big hospitals located in the city of Tehran (capital of Iran).

### Participants

The study population consisted of all nurses working at the two hospitals (N = 594). The sample size was calculated as 234 nurses based on Cochran’s formula at a confidence level of 95% and a significance level of 0.05. Considering the probability of sample dropout and questionnaires with incomplete information, the distributed questionnaires amounted to 30% more than the calculated sample size (n = 304).

In the inclusion criteria, we included having at least one year of work experience as a nurse, working in clinical wards, and possessing a Bachelor’s degree or higher, while for the exclusion criteria, we eliminated incomplete information in the questionnaires and also since the staff who do not have a bachelor are not categorized as nurses in Iranian hospital structures and are among other healthcare staff, we did not include them in the study.

### Data collection

The data collection tool for this research was a three-part questionnaire (demographic information and both WFC and ProQoL questionnaires). After obtaining the required permissions and coordinating with the selected hospitals, the researcher was referred to the hospitals for data collection. The questionnaires were delivered to the participants after providing them with sufficient information and obtaining their informed consent. As much as possible, the questionnaires were completed by the participants in the presence of the researcher. However, to prevent careless and hurried completion of the questionnaires, when nurses required additional time, the researcher returned to the hospitals later to collect the completed questionnaires.

### Questionnaires

#### Work-family conflict questionnaire

The WFC questionnaire by Carlson et al. [8] was used in this study and consisted of 18 questions exploring WFC in three different dimensions: time, strain, and behavior. There were six questions for each of the dimensions, and the questionnaire was scored based on a five-point Likert scale, ranging from strongly disagree (1) to strongly agree (5). The dimensions of the questionnaire included: time-based work-family conflict (the amount of time spent on work and less time spent on family), time-based family-work conflict (the amount of time spent on family activities and less time spent on work matters), work-family conflict based on the strain (that is, the amount of energy spent on work activities and reduces the energy spent on family matters), family- work conflict on the strain (that is, the amount of energy spent on family matters and reduces the energy spent on work activities), behavior-based work-family conflict (that is, the interference of work environment behaviors and norms with family behaviors and norms) and behavior-based family-work conflict (that is, the interference of family behaviors and norms with the behaviors and norms of the work environment), each they were measured using three questions.

Accordingly, the minimum and maximum obtainable scores were 18 and 90, respectively. More points indicate additional WFC. The validity of the questionnaire in the study was approved by Carlson et al. [5], and the reliability, ranging from 0.78 to 0.87, was measured and approved by Cronbach’s alpha coefficient for different dimensions. In a study by Motesharrei et al. [30] performed on nurses in hospitals in Shiraz, Iran, the questions were translated from English to Farsi, and the validity of the WFC questionnaire was further approved using the factor analysis method and calculating the fit index as 0.681. The reliability of the mentioned questionnaire was approved by obtaining a Cronbach’s alpha (0.91); therefore, we applied this study validated questions.

#### Professional quality of life questionnaire

The ProQoL questionnaire by Stamm [20] was used in this study. It consisted of 30 items and three subscales: compassion satisfaction, burnout, and stress subscale as the same as PTSD scale, with ten items for each subscale. A five-point Likert scale ranging from 1 (never) to 5 (always) was used for scoring the questionnaire.

In the sub-scale of satisfaction caused by sympathy, a score of 22 or less indicates low satisfaction, between 23 and 41 means moderate satisfaction, and a score of 42 and more indicates high sympathy satisfaction. A score of 22 or less in the second sub-scale indicates low job burnout, 23 to 41 indicates moderate job burnout, and a score of 42 or more indicates high job burnout. Finally, in the PTSD scale, a score of 22 or less indicates low stress, 23 to 41 indicates moderate stress, and 42 and above indicates high stress. The dimensions of this questionnaire are independent of each other, so it is impossible, to sum up the scores of the three sub-scales. As all dimensions of this questionnaire are independent of each other, adding the scores together is impossible. The validity of the ProQoL questionnaire was approved by Somoray et al. and its reliability was confirmed with Cronbach’s alpha of 0.90, 0.77, and 0.81 for compassion satisfaction, burnout, and PTSD, respectively [31].

#### Content and face validity of the ProQoL questionnaire

The questionnaire was first translated from original version to Persian and then both content validity and face validity of the Somoray questioners were assessed under the supervision of eight subject specialists (including epidemiologists and nursing experts). In addition, face validity was assessed using 10 nurses from the same region who did not participate into the main study. Internal consistency reliability for each scale was estimated: compassion satisfaction (ɑ = 0.85), burnout (ɑ = 0.82) and PTSD (ɑ = 0.84). The reliability was assessed by interviewing ten eligible people twice with a two-week period.

### Data analysis

SPSS software version 20 was used for data analysis by using descriptive and analytical statistics. Based on the results obtained from the Kolmogorov-Smirnov test, which showed a normal distribution of the variables (p > 0.05), the independent t-test was applied to compare the mean difference in the variables that had binary grouping such as gender, living with parents, and having a second job. One-way analysis of variance (One-way ANOVA) was used to compare the mean differences in variables with more than two categories, such as age, home ownership, and educational level. Pearson analysis was carried out to assess the correlation between variables of WFC and quality of professional life.

## Results

A nearly 81.9% response rate was obtained. From the total 249 collected questionnaires, 15 cases were omitted because of incomplete information, after which the data of the 234 analyzed cases were imported to the software. The demographic characteristics of participants are provided in Table 1.

**Table 1:** Demographic characteristics of participants

Characteristics		N (%)	Characteristics		N (%)
**Age (year)**	< 30 30–40 41–50 > 50	27 (11.5) 112 (47.9) 87 (37.2) 8 (3.4)	**Homeownership**	Owner Rental Government Leased Parent/relative	64 (27.4) 116 (49.6) 23 (9.8) 31 (13.2)
**Gender**	Male Female	129 (55.1) 105 (44.9)	**Educational level**	Bachelor’s Master’s/Doctorate	202 (86.3) 32 (13.7)
**Type of employment**	Permanent Contract Casual	170 (72.6) 54 (23.1) 10 (4.3)	**Marital status**	Single Married Divorced/widowed	53 (22.6) 165 (70.5) 16 (6.8)
**Economic status**	Bad Moderate Good	20 (8.5) 168 (71.8) 46 (19.7)	**Satisfactory relationship with colleagues**	Bad Moderate Good	48 (20.5) 138 (59.0) 48 (20.5)
**Living with parents**	Yes No	66 (28.2) 168 (71.8)	**Having a second job**	Yes No	29 (12.4) 205 (87.6)
**Having a disabled family member**	Yes No	15 (6.4) 219 (93.6)	**Monthly working hours**	< 175 ≥ 175	166 (70.9) 68 (29.1)
**Having a child less than one year old**	Yes No	14 (6.0) 220 (94.0)	**Having a close family member suffering from a chronic disease**	Yes No	31 (13.2) 203 (86.8)

The mean and standard deviation of the WFC scores are presented in Table 2. The total mean WFC was equal to 61.34, which is based on the possible range of 18 to 90, indicating a moderate condition. The highest and lowest means were related to WFC in terms of strain (23.05) and time (18.99), respectively.

**Table 2:** Work-family conflict (WFC) scores among participants

	Variables	Mean ± SD
**WFC**	WFC (time)	18.99 ± 1.79
	WFC (strain)	23.05 ± 1.54
	WFC (behavior)	19.28 ± 1.86
	Total	61.34 ± 4.60

Table 3 shows the mean and standard deviation as well as the level of ProQoL scores among participants. The highest mean was related to burnout (35.98), and more than 80% of nurses reported a moderate condition in terms of compassion satisfaction, burnout, and PTSD.

**Table 3:** Professional quality of life (ProQoL) scores and levels among participants

Variable		Mean ± SD	Low	Moderate	High
			**F (%)**	**F (%)**	**F (%)**
**ProQOL**	Compassion satisfaction Burnout PTSD	30.01 ± 3.60 35.98 ± 5.03 30.61 ± 3.34	4 (1.7) 0 (0.0) 0 (0.0)	230 (98.3) 197 (84.2) 234 (100.0)	0 (0.0) 37 (15.8) 0 (0.0)

An Independent t-test was used to measure the relationship between the demographic variables and the WFC and ProQoL scores (Table 4). The mean WFC and the three dimensions of ProQoL were higher among women than men (p < 0.05). The mean compassion satisfaction was lower among people who lived with their parents than those who did not (p = 0.036), and people who had a second job reported higher levels of burnout (p = 0.019). In terms of having a disabled family member, the findings showed that people who have a disabled family member have more unfavorable conditions than others in terms of WFC and burnout (p < 0.01). Also, the mean burnout was higher among nurses who had a child younger than one-year-old (p = 0.015), while they experienced less PTSD (p = 0.043). Finally, the findings showed that the mean WFC was higher among nurses who worked equal to or more than 175 h a month (p = 0.005).

**Table 4:** Association between WFC and ProQOL scores with demographic characteristics of participants

Characteristics	T test	WFC	Compassion satisfaction	Burnout	PTSD
**Gender**	t	−11.836	−3.459	−11.531	−2.281
	p-value	0.001*	0.001*	0.001*	0.023**
**Living with parents**	t	−0.278	−2.114	−0.457	−0.156
	p-value	0.781	0.036**	0.648	0.876
**Having a second job**	t	−1.165	−0.741	−2.366	0.838
	p-value	0.245	0.459	0.019**	0.403
**Having a disabled family member**	t	4.444	−0.019	4.657	0.698
	p-value	0.001*	0.958	0.001*	0.486
**Having a child less than one year old**	t	1.874	0.516	2.446	-2.040
	p-value	0.062	0.607	0.015**	0.043**
**Having a close family member suffering from a chronic disease**	t	1.016	−1.259	1.285	−1.447
	p-value	0.311	0.209	0.200	0.149
**Monthly working hours**	t	−2.805	1.446	−1.726	−1.342
	p-value	0.005*	0.149	0.086	0.181

*Difference statistically significant at 0.01 level, **Difference statistically significant at 0.05 level

The results of one-way ANOVA are presented in Table 5. Among the variables with significant relationships, the post-hoc test showed that the mean of WFC and burnout of nurses in the 30–40 and 41–50 age groups were significantly more than in the other groups (p < 0.05). Also, the mean of WFC and burnout among nurses living in rental houses was significantly more than for other nurses (p < 0.05). The results of the post-hoc test further demonstrated that the mean of burnout among permanent, contract, and casual employment was different so that the maximum and minimum values of this variable corresponded to permanent employed nurses and contract employed nurses, respectively (p < 0.05). Furthermore, the mean of WFC and burnout among single nurses was significantly lower than married and divorced/widowed nurses (p < 0.05). Finally, the results of the post-hoc test showed that the mean of WFC and burnout among nurses reporting the level of satisfying relationships with co-workers as ‘bad’ was significantly more than at the other levels (p < 0.05).

**Table 5:** Association between WFC and ProQOL scores with demographic characteristics of participants

Characteristics	ANOVA	WFC	Compassion satisfaction	Burnout	PTSD
**Age**	F	11.801	0.006	14.339	0.072
	p-value	0.001*	0.999	0.001*	0.975
**Home ownership**	F	6.221	1.585	4.963	0.816
	p-value	0.001*	0.194	0.002*	0.486
**Educational level**	F	0.079	0.351	1.482	1.248
	p-value	0.924	0.704	0.229	0.289
**Type of employment**	F	0.209	0.213	20.915	1.047
	p-value	0.821	0.808	0.001*	0.353
**Marital status**	F	9.305	0.148	8.224	0.440
	p-value	0.001*	0.862	0.001*	*0.645*
**Economic status**	F	1.479	1.459	1.721	0.231
	p-value	0.230	0.235	0.181	0.792
**Satisfactory relationship with colleagues**	F	10.011	1.532	11.424	0.781
	p-value	0.001*	0.218	0.001*	0.459

*Difference statistically significant at 0.01 level

The results of Pearson’s test regarding the correlation between WFC and ProQoL scores are provided in Table 6. The findings showed that there was a significant and positive relationship between WFC in terms of time and behavior with compassion satisfaction (p < 0.05). It was also observed that all types of WFC had a direct and significant relationship with burnout (p < 0.05), with the strongest observed correlation between WFC in terms of behavior and burnout. No significant relationship was observed between PTSD and the three types of WFC (p > 0.05).

**Table 6:** Correlation between the scores of WFC and ProQOL among nurses

	Pearson’s test	WFC in terms of time	WFC in terms of strain	WFC in terms of behavior
**Compassion** **satisfaction**	r	0.141	0.92	0.182
	p-value	0.031*	0.159	0.005*
**Burnout**	r	0.428	0.308	0.515
	p-value	0.001*	0.001*	0.001*
**PTSD**	r	-0.002	0.040	0.108
	p-value	0.971	0.541	0.101

*Difference statistically significant at 0.01 level

## Discussion

### The relationship between different levels of work-family conflicts and their sociodemographic characteristics

Importantly, avoiding WFC has a positive effect on nurses’ mental relaxation and their concentration for providing quality services to patients [32]. The findings of the present study showed that the nurses working at 2 hospitals in Tehran experienced a moderate level of WFC. Considering the WFC questionnaire of Carlson et al. in 2000 [5], which is applied vastly by various studies [5, 7, 21, 33, 34], the results of this study were consistent with other studies, specifically the results of a study in Jordan which revealed that the level of WFC among nurses was moderate [7]. Furthermore, in a study by Namayandeh et al. [22] in Shiraz, the mean of WFC was equal to 24.36 (in the range of 5–35), which shows a moderate level of WFC and is also consistent with our results [22]. However, the mean of WFC was 55.19 in a study by Fang in China [35]; despite being at a moderate level, it is numerically less than our results and indicates better conditions. Differences in the research environment that affect socio-cultural characteristics, as well as working conditions, can affect the incidence of these differences.

In this study, the mean of WFC in terms of time was at a moderate level. The level of this dimension in the study by Charkhabi et al. [34] in Iran was also moderate. Conversely, in a study by Alazzam et al. [7]in Jordan, this amount was higher than in our study, while the results of the study by Nutzi et al. [36] in Sweden indicated an ideal condition compared to our study [36]. Individual characteristics and workplace conditions could play a role in determining the level of WFC [37, 38].

The results of our study about WFC in terms of strain showed that this type of WFC had the highest mean among the three types of WFC and was in a moderate to high position. In Iran, it has become a long-standing problem that imposes excess pressure on nurses [39, 40]. Workforce shortages place high pressure on nurses, which due to the impact of high mental and physical stress, results in an inconsistency between work and personal life that ultimately inclines nurses to quit their job [41]. In a study by Hassanzadeh et al. [42], the level of WFC in terms of strain was less than in our study. As mentioned before, the conditions of the research environment could affect these results. Finally, evaluating WFC in terms of behavior showed that the mean value of this variable was at a moderate level in our study. A similar study in Greece obtained comparable results [43]. Our statistical tests revealed that the mean value of WFC among women was higher than men. Zurlo et al. also reported a higher mean value for WFC among women [44]. In addition to accepting some part of the financial responsibility for their family, working women play a crucial role in family activities such as taking care of the children and performing household chores [45]. In addition, female nurses scored higher than their male counterparts regarding the three dimensions of ProQoL: compassion satisfaction, burnout, and PTSD. Conversely, the study by Kim et al. [46] found there was no difference between female and male nurses in terms of the three dimensions of ProQoL [46]. Differences in socio-cultural conditions can be influential in this regard by influencing people’s attitudes and the status of women in society.

This study found no significant association between having a one-year-old or younger child with the level of WFC. However, we found a significant association between job burnout and having a younger child. Previous studies, however, have confirmed a link between having a child [47], especially a young child [48], and having a higher mean WFC. The reason may be that having a young child may create more responsibilities for parents, which, if not managed correctly and with no pre-planning, can have adverse effects on nurses’ jobs.

In terms of nurses’ marital status, the results revealed that the level of WFC and burnout among single nurses was less than that of married and widowed (divorced or passed away) nurses. The results of the study by Kim et al. [46] were consistent with our study and indicated that burnout in single nurses was less than in married ones. Regarding satisfaction with co-worker relationships, the statistical analysis demonstrated that the level of WFC and burnout among nurses reporting bad relationships with their co-workers was higher than others. In terms of the effect of co-worker relationships, the results of our study are consistent with the results of Hinderer et al. [49].

### The relationship between professional quality of life and its sociodemographic characteristics

Our findings revealed that the mean value for the compassion satisfaction dimension was at a moderate level. The reason for such satisfaction is feeling useful and valuable at work, combined with the existence of altruistic motivations [50]. The results of studies by Sacco et al. [51] in the USA, Hegney et al. [52] in Australia, and Circenis et al. [53] in Latvia all showed mean values for their compassion satisfaction dimension that were higher than the value obtained from our study. All individual and work factors influencing nurses’ precision and quality of performance can affect compassion satisfaction [54]. One of the main factors in this regard is the shortage of nurses in Iran. Recently Iran has faced a shortage of 100,000 nurses [39, 40].

The mean value for burnout in this study was at a moderate level. Evaluating the frequency distribution of participants in terms of the level of burnout revealed that 84.2% of them experienced a moderate level of burnout. Compared with the findings of other studies, nurses in our study experienced a better situation regarding burnout [52, 53]. Different factors such as personal characteristics, personal and family life, work, and workplace factors, as well as a society’s economic and cultural conditions, can also affect burnout [55, 56]. Thus, observing the contradiction among different studies is not surprising due to the differences between these factors in various societies and research environments.

The last dimension among the three dimensions of ProQoL is PTSD, which obtained a moderate level in this study. PTSD in nurses can cause adverse effects on the quality of service provided to patients and is introduced as a predisposing factor for burnout. Those two mentioned outcomes can be influenced by workload. Heavy workloads increase the possibility of facing stressful conditions, which causes a relapse of PTSD [57]. Cimiotti et al. [33] suggested that reducing burnout among US nurses by 10% could prevent 4,150 hospital infections and save up to 41 million US dollars, annually. In Taiwan, Tseng et al. [27] reported worse conditions in terms of PTSD among nurses, such that those nurses reported a higher mean value for PTSD compared with our study [27].

The obtained results also demonstrated that burnout as one of the dimensions of ProQoL has a direct correlation with the three dimensions of conflict: time, strain, and behavior, with the strongest correlation, observed between burnout and behavior. In a study by Kleiner et al. [58], however, conflict in terms of time was defined as the most critical determinant of burnout. Similarly, in the Greece study, WFC caused an increase in burnout [43]. The relationship between WFC and burnout was indeed mutual, as these two variables mutually influence each other.

### Strengths and limitations

The main limitation of this study is that the survey was conducted in two hospitals in Tehran (The capital of Iran), and the results may not be generalizable to hospital nurses in other systems, such as public, private, or university hospitals, and to other geographic areas. Finally, given the cross-sectional nature of our study, causality was not determined.

### Practical implication

The findings demonstrate that different strategies should be established by nurse administrators and policymakers to support the balance between the nurses’ family life and nurses’ work life, including child care services and other liomofe benefits. Hospitals need to improve their work environments that increase the job satisfaction of nurses, which leads to increasing patients’ satisfaction and the quality of nursing care services.

## Conclusion

The mean WFC among the studied nurses was at a moderate level. WFC can influence the quality of nurses’ performance and job satisfaction and may also result in nurses intending to leave their jobs. Correspondingly, the means of burnout and PTSD in this study were at a moderate level, and these factors can affect patient outcomes in hospitals. Furthermore, stable mental and emotional conditions are of great importance for nurses to provide safe and quality services to their patients. The results also revealed that nurses’ compassion satisfaction and job burnout were associated with different types of conflict. The WFC of nurses is related to their ProQoL and affects their job satisfaction and burnout. Reducing conflict may improve the nurses’ satisfaction and thus improve patient care and healthcare services.

## Data Availability

The datasets generated and analyzed during the current study are not publicly available due to these data were used under license for the current study but are available from the corresponding author on reasonable request.

## Abbreviations

WFC: Work-family conflict
ProQoL: Professional quality of life
PTSD: Post-traumatic stress disorder
